# Phylogenetic insights based on the first complete mitochondrial genome of *Isomyia nebulosa* (Diptera: Calliphoridae)

**DOI:** 10.1080/23802359.2023.2288916

**Published:** 2023-12-04

**Authors:** Ting Ma, Caihong Zhang, Jia Huang

**Affiliations:** a Police Experimental Training Center, Guangdong Police College, Guangzhou, Baiyun, China; b Department of Entomology, College of Plant Protection, South China Agricultural University, Tianhe, Guangzhou, China

**Keywords:** Forensic entomology, mitogenome, Oestroidea, phylogeny, Rhiniinae

## Abstract

To investigate the phylogenetic position of *Isomyia* Walker, 1860, a genus that suffered from frequent revisions of the taxonomic status following the subfamily Rhiniinae (Diptera, Calliphoridae), we sequenced, assembled, annotated, and analyzed the first complete mitochondrial genome (mitogenome) of *Isomyia nebulosa* (Townsend, 1917) in this study. This mitogenome is 16,438 bp in length, with a standard set of 13 protein-coding genes (PCGs), 22 tRNAs, two rRNAs, and an A + T riched non-coding region without genetic rearrangement as most dipteran mitogenomes, but long intergenic nucleotides (IGNs) between *trnQ* and *trnM* are found. The phylogeny yielded by both Bayesian inference and maximum-likelihood analysis for all mitochondrial PCGs and rRNAs of 23 mitogenomes supports the monophyly of the family Calliphoridae and the subfamilies Calliphorinae, Chrysomyinae, and Luciliinae. In addition, *I. nebulosa* diverged anterior to the above-mentioned three calliphorid subfamilies with high genetic distances.

## Introduction

Due to the metallic appearance and sarcosaprophagy, the members of the genus *Isomyia* Walker, 1860 are particularly similar to those common blow flies in the family Calliphoridae and are considered to have medical importance and forensic potential (Yan et al. 2021). The *Isomyia* has long been classified as the largest genus in the subfamily Rhiniinae of the Calliphoridae (Heo et al. 2013; Bunchu et al. 2014; Thomas-Cabianca et al. 2023). Benefiting from the rapid development of molecular biology and sequencing technologies, the phylogenetic framework within the superfamily Oestroidea has caused widespread controversy in recent years, leading to frequent revisions of the taxonomic status of the Rhiniinae (Marinho et al. 2012; Singh and Wells 2013; Yan et al. 2021). However, the *Isomyia* is currently inclined to be regarded as a genus of unassigned subfamily in the Calliphoridae in taxonomy (Bánki et al. 2023), on account of the lack of sampling of *Isomyia* species in related molecular phylogenetic studies.

In this study, we sampled *Isomyia nebulosa* (Townsend, 1917), a common *Isomyia* species distributed in South Asia (southwestern China, India, and Myanmar), obtained its complete mitochondrial genome (mitogenome) sequence (NCBI accession number OR497843), and performed a phylogenetic analysis for exploring its phylogenetic position and the calliphorid monophyly using available mitogenomes.

## Materials and methods

**Figure 1.**
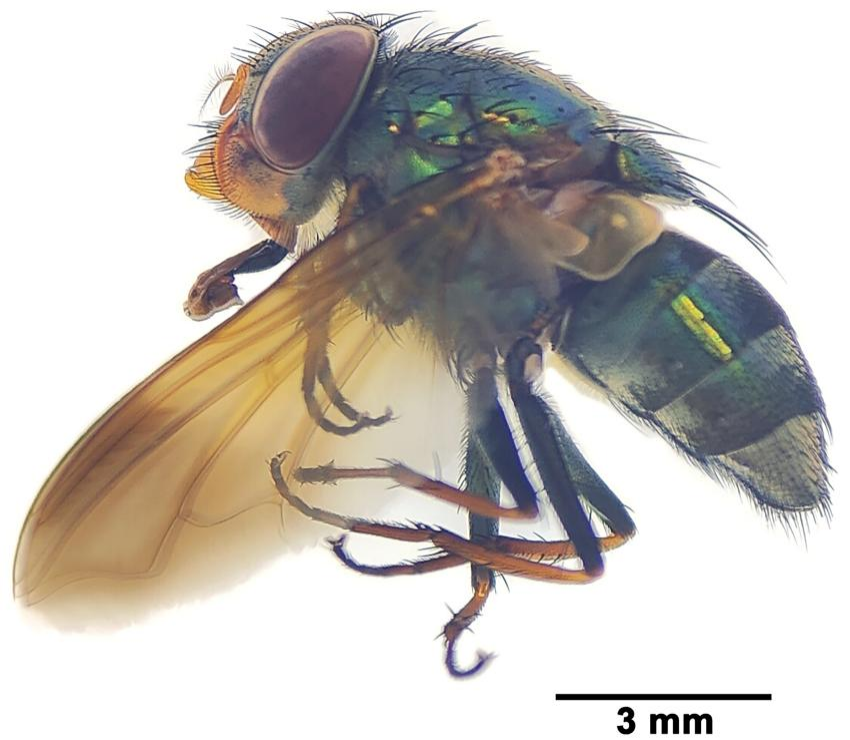
Reference image of female *Isomyia nebulosa* (Townsend, 1917) collected from Cangyuan, Lincang, Yunnan, China. This image was photographed by Jia Huang.

We collected three female adults (Figure 1) around a snake carcass in Cangyuan, Lincang, Yunnan, China (23°54′28ʺN, 99°13′52ʺE, this location was open and no permission was required for insect collection) in May 2016. Jia Huang identified this species using systematic morphological identification keys of the Calliphoridae (Fan et al. 1997). *I. nebulosa* is characterized by the dark pattern on the distal part of wing (Figure 1) in the *dotata* species group Peris, 1952. All of these specimens were deposited at the Department of Entomology (Jia Huang, hj@scau.edu.cn), South China Agricultural University (SCAU), Guangzhou, China under the voucher number (H000041–43). The total DNA of one of the specimens was extracted from its thoracic muscles using the HiPure Tissue DNA Mini Kit (#D3121, Magen Biotech., Guangzhou, China) following the manufacturer’s protocol. Beijing Geneplus Technology Co., Ltd. (China) performed DNA Nano Ball (DNB) library construction and quality control for the provided DNA sample. The main steps of the DNB library construction included denaturation, single-strand circularization, exonuclease digestion, and DNB amplification. The constructed library was loaded on a DNBSEQ-T7 platform (BGI, Shenzhen, China) and sequenced with 2 × 150 bp paired-end (PE150) mode.

The mitogenome of *I. nebulosa* was assembled from the raw sequencing data (NCBI Short Read Archive, SRA accession number SRR25915118) using Geneious Prime v2023.0.4 and annotated following the study of Zhang et al. (2023) except for using the mitogenome of *Polleniopsis mongolica* Séguy, 1928 (MT017721) (Shang et al. 2022) as a reference. We depicted the circular mitogenome using the Proksee server (Stothard et al. 2019). Its nucleotide composition was calculated in MEGA v7.0.26 (Kumar et al. 2016).

In this study, we employed the other 22 complete or almost complete mitogenomes for subsequent phylogenetic analyses (Table 1), including 18 Calliphoridae species, two Sarcophagidae species (as outgroup taxa in the superfamily Oestroidea), and two Muscidae species (as outgroup taxa in the Calyptratae). Although some genera (i.e. the *Calliphora*, *Chrysomya*, and *Lucilia*) have more available mitogenomes in NCBI, only two representatives of each of them were selected due to their mitogenomic monophyly has been well-supported in previous studies (e.g. Shang et al. 2022) and reduced sampling in our analyses will not change the topology of the final trees. The nucleotide sequences of concatenated 13 protein-coding genes (PCGs) and two rRNAs of the 23 mitogenomes were aligned, partitioned, analyzed, and visualized following the methods in Zhang et al. (2023). Briefly, we used PartitionFinder v2.1 (Lanfear et al. 2017) for selecting best-fit partitioning schemes and models, and used MrBayes v3.2.7a (Ronquist et al. 2012) for Bayesian inference (BI) and IQ-TREE v2.2.2.7 (Minh et al. 2020) for maximum likelihood (ML) analysis.

**Table 1.** Details of the mitogenomes used in the phylogenetic reconstructions.

Family	Tribe	Genus	Species	Mitogenome length (bp)	NCBI accession number	Reference
Calliphoridae	Bengaliinae	*Bengalia*	sp.	15,748, complete	MK591038	Tang et al. 2019
Calliphoridae	Calliphorinae	*Calliphora*	*vicina* Robineau-Desvoidy, 1830	16,112, complete	JX913760	Nelson et al. 2012
Calliphoridae	Calliphorinae	*Calliphora*	*vomitoria* (L., 1758)	16,134, complete	KT444440	Ren et al. 2016
Calliphoridae	Calliphorinae	*Cynomya*	*mortuorum* (L., 1761)	15,003, partial	MT628574	Unpublished (submitted by Leerhoei, F.)
Calliphoridae	Calliphorinae	*Polleniopsis*	*mongolica* Séguy, 1928	15,647, complete	MT017721	Shang et al. 2022
Calliphoridae	Chrysomyinae	*Chloroprocta*	*idioidea* (Robineau-Desvoidy, 1830)	14,912, partial	KT272777	Junqueira et al. 2016
Calliphoridae	Chrysomyinae	*Chrysomya*	*albiceps* (Wiedemann, 1819)	15,491, complete	JX913736	Nelson et al. 2012
Calliphoridae	Chrysomyinae	*Chrysomya*	*megacephala* (Fabricius, 1794)	15,273, complete	JX913738	Nelson et al. 2012
Calliphoridae	Chrysomyinae	*Cochliomyia*	*hominivorax* (Coquerel, 1858)	16,022, complete	AF260826	Lessinger et al. 2000
Calliphoridae	Chrysomyinae	*Cochliomyia*	*macellaria* (Fabricius, 1775)	14,924, partial	KT272853	Junqueira et al. 2016
Calliphoridae	Chrysomyinae	*Hemilucilia*	sp.	14,898, complete	KT272860	Junqueira et al. 2016
Calliphoridae	Chrysomyinae	*Paralucilia*	sp.	14,859, partial	KT272861	Junqueira et al. 2016
Calliphoridae	Chrysomyinae	*Phormia*	*regina* (Meigen, 1826)	15,513, complete	KC005712	Ramakodi et al. 2015
Calliphoridae	Chrysomyinae	*Protocalliphora*	*azurea* (Fallén, 1817)	15,706, complete	OW026526	Falk and Sivell 2023
Calliphoridae	Chrysomyinae	*Protophormia*	*terraenovae* (Robineau-Desvoidy, 1830)	15,170, complete	JX913743	Nelson et al. 2012
Calliphoridae	Luciliinae	*Hemipyrellia*	*ligurriens (Wiedemann, 1830)*	15,938, complete	JX913759	Nelson et al. 2012
Calliphoridae	Luciliinae	*Lucilia*	*porphyrina* (Walker, 1856)	15,877, complete	JX913758	Nelson et al. 2012
Calliphoridae	Luciliinae	*Lucilia*	*sericata* (Meigen, 1826)	15,945, complete	AJ422212	Stevens et al. 2008
Calliphoridae	Rhiniinae	*Isomyia*	*nebulosa* (Townsend, 1917)	16,438, complete	OR497843	This study
Muscidae	Muscinae	*Musca*	*domestica* L., 1758	16,108, complete	KM200723	Li et al. 2016
Muscidae	Muscinae	*Musca*	*sorbens* Wiedemann, 1830	16,120, complete	MG941012	Ma and Huang 2018
Sarcophagidae	Sarcophaginae	*Sarcophaga*	*dux* Thomson, 1869	15,731, complete	MH540745	Huang and Ma 2018
Sarcophagidae	Sarcophaginae	*Sarcophaga*	*peregrina* (Robineau-Desvoidy, 1830)	14,922, complete	KF921296	Zhong et al. 2016

## Results

### Mitogenomic characteristics

**Figure 2.**
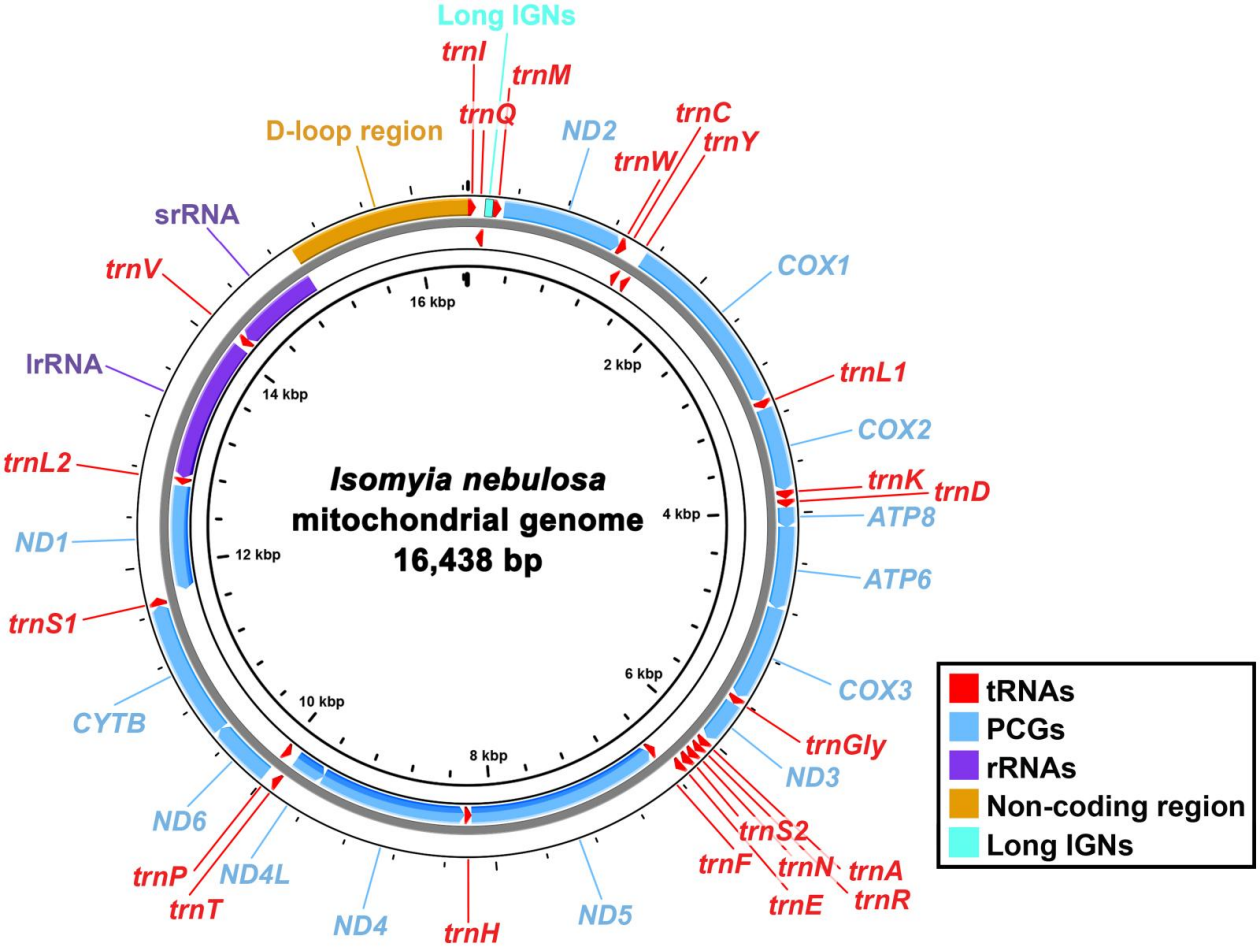
Graphical map of the complete mitochondrial genome (mitogenome) of *I. nebulosa* assembled in this study. Arrows indicate the directions of transcription.

The complete *I. nebulosa* mitogenome assembled in this study is 16,438 bp in length (Figure 2), with 1,173 to 40,076× coverage depth (supplementary material: Figure S1). This is currently the sixth-largest mitogenome among 65 known calliphorid mitogenomes in NCBI. It contains the typical set of 37 genes (13 PCGs, 22 tRNAs, and two rRNAs) and an A + T riched non-coding region without genetic rearrangement. The lengths of the PCGs, tRNAs, and rRNAs range from 165 (*ATP8*) to 1,738 bp (*ND5*), 62 (*trnR*) to 77 bp (*trnV*), and 785 (srRNA) to 1,300 bp (lrRNA), respectively. Twelve PCGs share start codon ATD (ATA for *ND1*; ATG for *ATP6*, *COX2*, *COX3*, *CYTB*, *ND4*, and *ND4L*; ATT for *ATP8*, *ND2*, *ND3*, *ND5*, and *ND6*), whereas *COX1* starts with TCG as other dipteran species (Huang and Ma 2018; Zhang et al. 2023). All PCGs terminate with either TAY (TAA for *ATP6*, *ATP8*, *COX2*, *COX3*, *CYTB*, *ND2*, *ND3*, *ND4L*, and *ND6*; TAG for *ND1*) or incomplete stop codon T (*COX1*, *ND4*, and *ND5*). The intergenic nucleotides (IGNs) of *I. nebulosa* mitogenome spread over 18 pairs of adjacent genes and range in size from 1 to 71 bp with a total length of 263 bp. The longest IGNs have high A + T content and are located between *trnQ* and *trnM* (Figure 2). Similarly, a total of seven overlapping regions ranging from 1 bp to 8 bp with a total length of 20 bp are identified in this mitogenome. The nucleotide composition of its majority strand is computed as *A* = 40.82%, *T* = 38.32%, *G* = 8.46%, and *C* = 12.40%, as a consequence, with a significant bias toward A and T (79.14%) especially in the non-coding region (87.67%).

### Phylogenetic analyses

**Figure 3.**
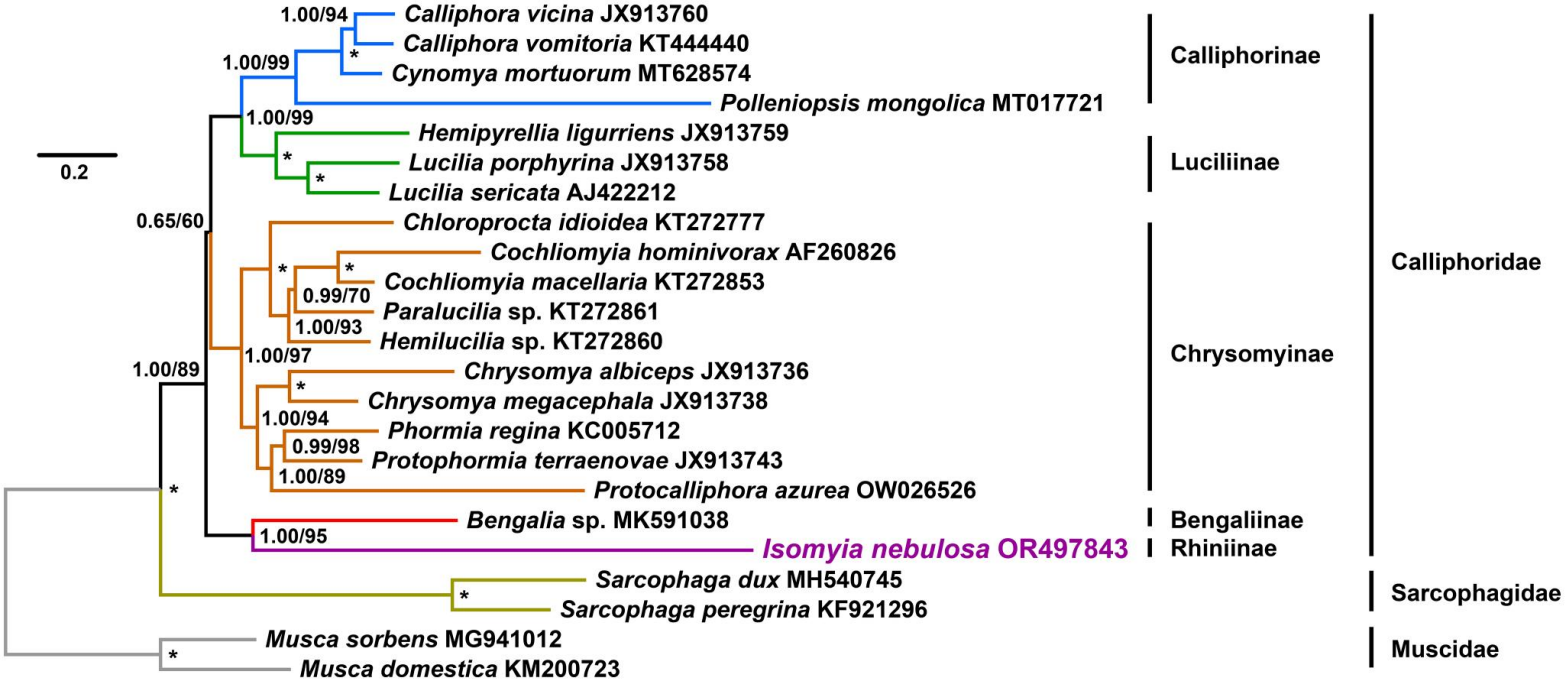
Phylogenetic tree reconstructed based on all mitochondrial protein-coding genes (PCGs) and rRNAs of 23 mitogenomes by Bayesian inference (BI). numbers around nodes are posterior probabilities (PPs, left) of the BI and ultrafast bootstrap percentages (UBPs, right) inferred from the maximum likelihood (ML) analysis. Asterisks (*) indicate full support (PP = 1.00, UBP = 100). the bar indicates the estimated number of substitutions per site.

In this study, we performed both BI and maximum likelihood analysis for the 15-gene dataset of 23 mitogenomes (Table 1). Overall, the final consensus trees show an entirely identical topology (Figure 3). Nineteen of 21 nodes are well-supported in both analyses (posterior probabilities, PPs = 1.00, ultrafast bootstrap percentages, UBPs ≥ 89), indicating the mitogenomic monophyly of the families Calliphoridae (PP = 1.00, UBP = 89), Muscidae (PP = 1.00, UBP = 100), and Sarcophagidae (PP = 1.00, UBP = 100), and the monophyly of the subfamilies Calliphorinae (PP = 1.00, UBP = 99), Chrysomyinae (PP = 1.00, UBP = 97), and Luciliinae (PP = 1.00, UBP = 99). At the subfamilial level, our analyses highly support the close relationship between the Calliphorinae and Luciliinae (PP = 1.00; UBP = 99), but the subfamilial relationship between the Calliphorinae + Luciliinae and the Chrysomyinae is not well-supported in both trees (PP = 0.65, UBP = 60). The subfamily Rhiniinae represented by the *I. nebulosa* mitogenome shows a closed relationship with the subfamily Bengaliinae represented by a *Bengalia* species (PP = 1.00, UBP = 95). Both trees show the Bengaliinae + Rhiniinae diverged anterior to the three common calliphorid subfamilies (PP = 1.00; UBP = 89). In addition, all congeneric mitogenomes are also firmly supported as monophyletic as expected (PPs = 1.00, UBPs ≥ 94).

## Discussion and conclusion

In this study, we provided and analyzed the first complete mitogenome of *I. nebulosa* as a representative for the Rhiniinae. This newly assembled mitogenome contains the typical mitogenomic gene set as most dipteran species (Zhang et al. 2015; Huang and Ma 2018). Our study clearly demonstrates the phylogenetic placement of the Rhiniinae relative to the Calliphoridae based on mitogenomes. On the other hand, the *I. nebulosa* mitogenome shows significant genetic divergences from the other calliphorid mitogenomes with interspecific *p*-distances ranging from 10.75 [*Lucilia sericata* (Meigen, 1826)] to 12.00% [*Lucilia porphyrina* (Walker, 1856)]. It can be confirmed that this mitogenome plays a significant role in defining the clear boundary of the Calliphoridae. The *I. nebulosa* mitogenome assembled in this study, along with more available mitogenomes of other related taxa, will contribute to a clearer understanding of the phylogenetic framework within the Oestroidea.

## Supplementary Material

Supplemental Material

## Data Availability

The mitogenome sequence that supports the findings of this study is openly available in NCBI (https://www.ncbi.nlm.nih.gov/) under the accession number OR497843. The associated BioProject, SRA, and BioSample accession numbers are PRJNA1010982, SRR25915118, and SAMN37205466, respectively.
