# Supplementary material for: Phylogenetic insights based on the first complete mitochondrial genome of Isomyia nebulosa (Diptera: Calliphoridae)
Source: Mitochondrial DNA B Resour. 2023 Dec 4;8(12):1320–4. doi: 10.1080/23802359.2023.2288916 (PMC10776078; doi:10.1080/23802359.2023.2288916)
Supplement: Supplemental Material [file TMDN_A_2288916_SM5848.pdf]

Supplemental material:

Phylogenetic insights based on the first complete mitochondrial genome of *Isomyia nebulosa* (Diptera: Calliphoridae)

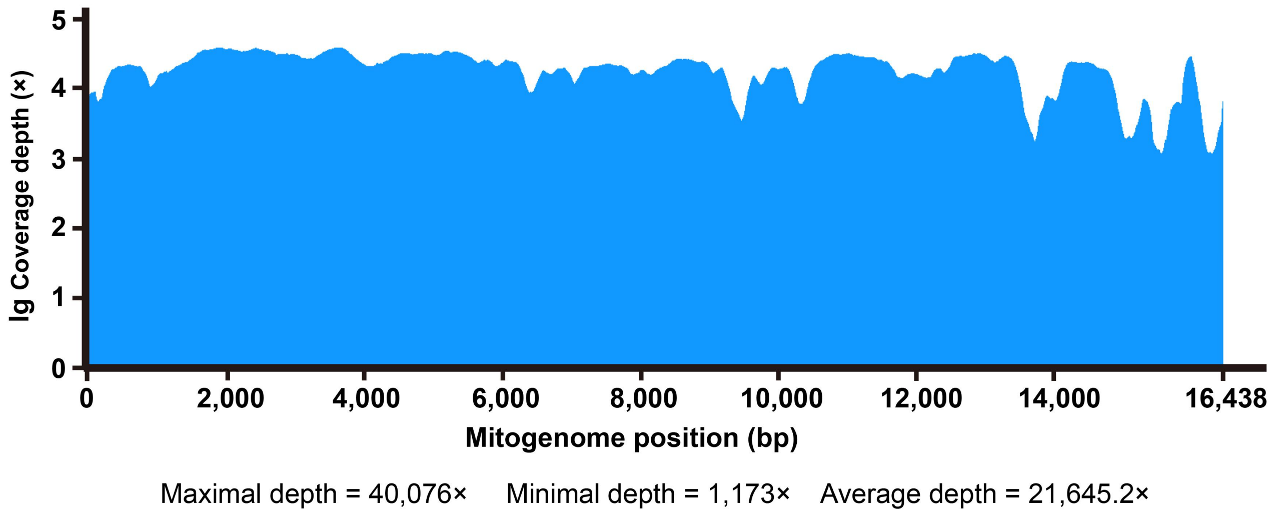

Figure S1. Coverage depth plot of the mitochondrial genome (mitogenome) of *Isomyia nebulosa* assembled in this study. X-axis and Y-axis show nucleotide position and coverage depth of *I. nebulosa* mitogenome, respectively. We generated this plot by mapping the raw DNA sequencing data (SRA accession number SRR25915118) to the mitogenome (NCBI accession number OR497843) using Geneious Prime v2023.0.4 without mismatch base (maximum mismatches per read = 0%).
